# Mathematical description data: Spin-resolved electron transport in nanoscale heterojunctions: Theory and applications

**DOI:** 10.1016/j.dib.2020.106233

**Published:** 2020-09-01

**Authors:** Artur Useinov, Hsiu-Hau Lin, Niazbeck Useinov, Lenar Tagirov

**Affiliations:** a International College of Semiconductors Technology, National Chiao Tung University, Hsinchu 30010, Taiwan; b Department of Physics, National Tsing Hua University, Hsinchu 30013, Taiwan; c Institute of Physics, Kazan Federal University, Kazan 420008, Russian Federation; d Zavoisky Physical-Technical Institute, FRC Kazan Scientific Center of RAS, Kazan 420029, Russian Federation; e Tatarstan Academy of Sciences, Institute of Applied Research, Kazan 420111, Russian Federation

**Keywords:** Ballistic and diffusive transport model, Point-like contact model, Spin-resolved contact conductance, Heterojunctions, *I–V modeling*

## Abstract

This study demonstrates a mathematical description of a point-like nanocontact model, which is developed to simulate electron transport through a nanoconstriction between magnetic or non-magnetic contact sides. The theory represents a solution to the quasi-(semi)-classical transport equations for charge current, which takes into account second-order derivatives of the related quasi-classical Green functions along the transport direction. The theoretical approach also enables the creation of an *I–V* model for a heterojunction with embedded objects, where the initial condition, a conduction band minimum profile of the system, is well-defined. The presented spin-resolved current approach covers a complete range of the scales including quantum, ballistic, quasi-ballistic (intermediate), and diffusive classical transport conditions, with a smooth transition between them without residual terms or any empirical variables. The main benefit of the mathematical solution is its novel methodology, which is an alternative candidate to the well-known Boltzmann technique.

## Specifications Table

**Table:** 

Subject	Nanotechnology, modeling and simulation, applied mathematics, and metals and alloys
Specific subject area	The mathematical solution of the quasi-classical transport equations that describe electron transport through a nanocontact valid from a Maxwell diffusive conductance to a ballistic one without residual terms.
Type of data	Series of intermediate equations
How data were acquired	Mathematical analysis of the integro-differential equations was developed to derive the most accurate solution. Wolfram Mathematica was partly applied as an analytical programming language.
Data format	Enhanced Model proof Raw
Data collection parameters	A mathematical solution of the integro-differential equation for the electron transport takes into account the second-order derivatives of the Green functions along a transport direction.
Description of data collection	The extension of the theoretical model describes spin-resolved electron transport in nanoscale magnetic contacts and heterojunctions.
Data source location	Institution: National Chiao Tung University City/region: Hsinchu/ Hsinchu Country Country: Taiwan
Data accessibility	Repository name: Mendeley repository data Data identification number: doi: 10.17632/t868bx922b.4 Direct URL to data: https://data.mendeley.com/datasets/t868bx922b/4
Related research article	A. Useinov, H.H. Lin, N. Useinov, L.Tagirov, Spin-resolved electron transport in nanoscale heterojunctions. Theory and applications, Journal of Magnetism and Magnetic Materials 508 (2020) 166,729. https://doi.org/10.1016/j.jmmm.2020.166729

## Value of the Data

• The presented data make it possible to construct the *I-V* characteristics of point junctions and heterostructures applicable on a scale of 0.5–400 nm (and above) since the model combines Maxwell's diffusion, intermediate, and ballistic transport conditions without residual terms. Quantum boundary conditions are used for the interface boundaries.
• The mathematical algorithm and methods as the advantages of the presented solution can serve as useful examples for mathematicians and physicists attempting to solve transport or diffusion problems with boundary conditions.
• The model is potentially oriented toward further improving contact Andreev reflection and point-contact spectroscopies, modeling magnetic heterojunctions, and scanning tunneling microscopy methods.
• The model allows a more accurate fit of a wide range of experimental data on non-magnetic symmetrical point contacts.
• The model enables the simulation of the conductance (resistance) of magnetic point contacts.

## 1 Data Description

**Fig. 1:**
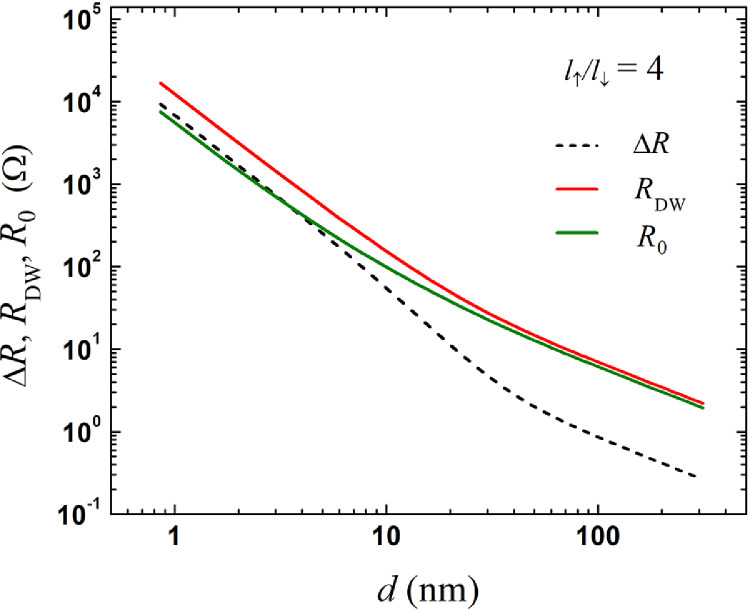
Logarithmic scale of the magnetic point contact (MPC) resistance with (*R*_DW_) and without (*R*_0_) a single DW *vs* the MPC's diameter, d=2a, also showing the difference between them, $\Delta R=R_{\text{DW}}-R_{0}$. The model's parameters: $l_{↑}/l_{↓}=4$, $l_{↑}=12.0$nm, $l_{↓}=3.0$nm, $k_{F↑}^{L}=0.61\;{\text{Å}}^{-1}$, $k_{F↓}^{L}=1.08\;{\text{Å}}^{-1}$, $k_{F↑}^{R}=0.61\;{\text{Å}}^{-1}$ and $k_{F↓}^{R}=1.08\;{\text{Å}}^{-1}$ for a DW 3.0 nm wide.

**Fig. 2:**
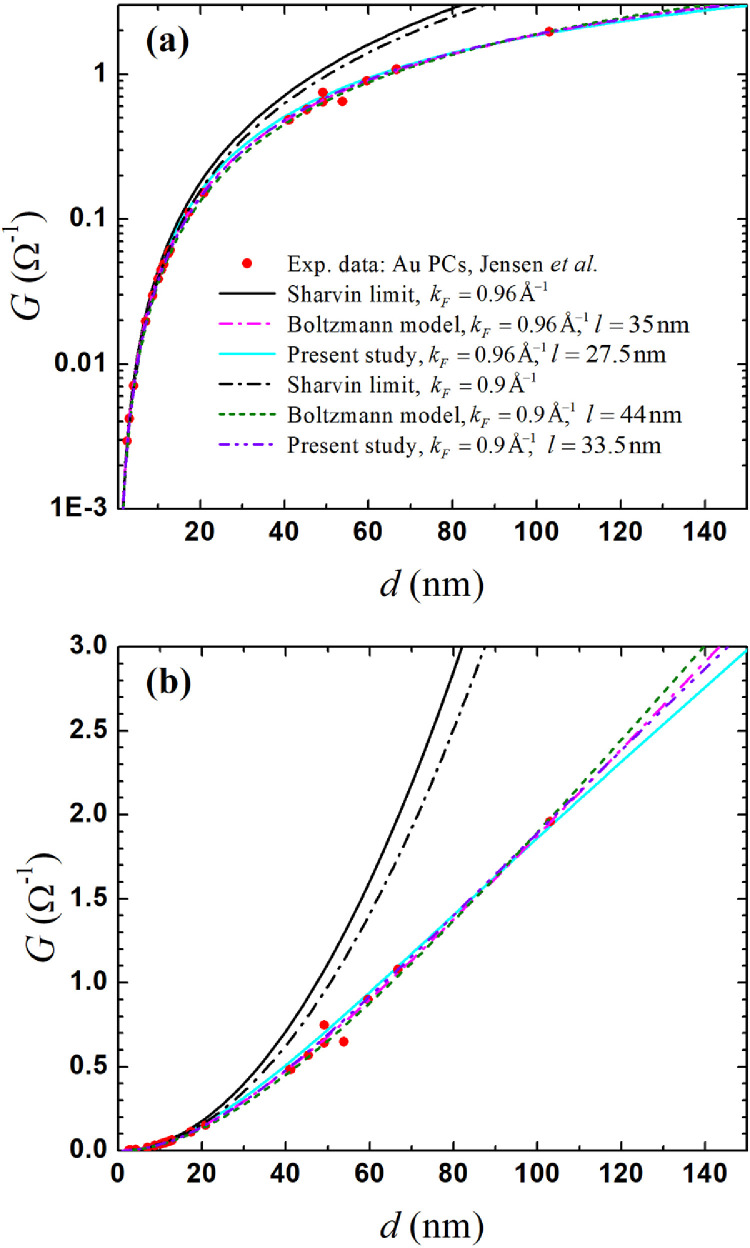
Point contact conductance *vs* diameter in logarithmic (**a**) and linear (**b**) scales. The red solid circles are experimental points for the golden PCs that were redrawn from Fig. 4 in Ref. [10]. The magenta dash-dot and green dashed curves correspond to the Boltzmann-based model in Ref. [11]. The legend in (**a**) refers equally to (**b**).(For interpretation of the references to color in this figure legend, the reader is referred to the web version of this article.)

The theoretical model presents a mathematical technique and methodology for solving the problem of electron transfer in a nanoscale point contact (PC). The model is formulated in terms of kinetic equations for symmetric and asymmetric Green functions (GF), which are responsible for the difference in the chemical potentials and charge current density through the contact, respectively. The transition to cylindrical coordinates and the Fourier transform enables the presentation of a formally exact solution of the equations in an integral form as well as the expansion of integrands along a quasi-classical trajectory in a series up to the second-order transformation of integral equations to a simpler form. To solve the asymmetric GF and the associated current density, the equations obtained are averaged over the solid angle of the electron trajectory in spherical coordinates, demonstrating the relationship between the symmetric and asymmetric GF on each side of the contact. A solution to the equations for average GFs with applied Zaitsev boundary conditions adapted for the ferromagnetic contact interface obtains a universal expression of the point contact conductance of two generally heterogeneous ferromagnetic metals. As a first example of the general theoretical model's application, the single domain wall (DW) impact on the resistance of the magnetic point contact is estimated and presented in Fig. 1. As a second application, the conductance of a particular case of nanocontacts made of non-magnetic metal was calculated (Fig. 2) and compared with Boltzmann model, a novel and rather simple expression was obtained that describes a continuous transition from the diffuse mode of electron transport through the nanocontact to the ballistic one without redundant terms in early solutions to this problem. The program builder for Fig. 2 is enabled in Supplementary Materials.

## 2 Theoretical Methods and Applications

### 2.1 Theoretical model (methods)

A mathematical model of a junction with point contact (PC) geometry is considered. The contact area itself is constricted with a circular cross-section and connects large left and right reservoirs of electron gas. The parameter that quantifies the PC's conductance conditions is the dimensionless ratio *a*/*l*, where *l* is the bulk electron mean free path (mfp) and *a* is the radius of the contact area. The model has spherical [**k**, *θ*, φ] and aligned cylindrical [**r**, *ϕ, z*] coordinate systems, and the *z* axis is perpendicular to the PC plane (PCP), as shown in Fig. 1 in [1]. An applied voltage *V* induces the current $I^{z}=I_{↓}^{z}+I_{↑}^{z}$. The current density $j_{\alpha }^{z}$ of the spin index *α* is expressed as follows according to [2]:

(1) $$j_{\alpha }^{z}\left(z\to 0,k,\varepsilon \right)=-\;\frac{e\;k_{F,\alpha }^{2}}{2\pi \;\hbar }\langle \cos\theta_{\alpha }\;g_{a,\alpha }\left(z,k,\varepsilon \right)\rangle ,$$

where ɛ, **k**, and *θ_α_* are the electron energy, its wavevector, and the angle between the electron's quasi-classical path and the *z* axis, respectively. The expression 〈(..)〉=∮(..)dΩ/2π denotes the solid angle average over Ω. The variable *g*_*a, α*_(*z*, **k**, ɛ) is a Green function (GF). A Fourier transform of the GF in Eq. (1) is:

(2) $$g_{a\;(s),\alpha }\left(z,r,t\right)=\int \frac{d^{2}k}{{\left(2\pi \right)}^{2}}\frac{d\varepsilon }{2\pi }g_{a(s),\alpha }\left(z,k,\varepsilon \right)\;e^{-i\left(k\;r-\varepsilon \;t\right)},$$

where $g_{a(s),\;\alpha }\left(z,r,t\right)=\frac{1}{2}\left[g_{\alpha }\left(n_{z},z,r\right)\mp g_{\alpha }\left(-n_{z},z,r\right)\right]$ is the antisymmetric (symmetric) GF with respect to the projection on the *z* axis of the Fermi momentum $n_{z}=p_{z\;,\alpha }/p_{F,\alpha }$ and **r**is located in the center of the PCP radius vector.

The stationary charge current is expressed in terms of the current density in a mixed representation of Eq. (1) integrated over **r** in a cylindrical coordinate system at *z* → 0:

(3) $$I_{\alpha }^{z}\left(z\to 0\right)=a\;\int_{0}^{\infty }J_{1}\left(ka\right)j_{\alpha }^{z}\left(z,k\right)\;dk,$$

where *J*_1_(*ka*) is the Bessel function appearing due to integration over the circular PCP. The GF *g_a_*(*z*, **r**, *t* → 0) is a solution to the kinetic equations along the quasi-classical trajectories formulated by Tagirov et al. [2,3] in the form similar to Eq. (4). The electron transport in terms of GFs can be expressed for quantum and classical PC junctions utilizing the following equations:

(4) $$\begin{array}{c} l_{z,\alpha }\frac{\partial g_{a,\alpha }\left(z,r\right)}{\partial z}+l_{∥,\alpha }\frac{\partial g_{s,\alpha }\left(z,r\right)}{\partial r}+g_{s,\alpha }\left(z,r\right)-\langle g_{s,\alpha }\left(z,r\right)\rangle =0,\\ l_{z\;,\alpha }\frac{\partial g_{s,\alpha }\left(z,r\right)}{\partial z}+l_{∥,\alpha }\frac{\partial g_{a,\alpha }\left(z,r\right)}{\partial r}+g_{a,\alpha }\left(z,r\right)=0, \end{array}$$

where $l_{∥,\alpha }=\tau v_{∥,\alpha }^{F}$ is the spin-dependent mfp projection on the PCP. The modulus of the vector is $l_{∥,\;\alpha }=\sqrt{l_{\alpha }^{2}-l_{z,\alpha }^{2}}$, where $l_{z,\alpha }=l_{\alpha }\cos\left(\theta_{\alpha }\right)=v_{z}\tau$ is a projection on the *z* axis. The brackets ${\langle ...\rangle }_{\theta_{c}}$ determine the average over the solid angle ${\langle ...\rangle }_{\theta_{c}}=\frac{1}{2\pi }\int_{0}^{2\pi }d\varphi \int_{0}^{\pi /2}\left(..\right)\;\;\sin\left(\theta_{c}\right)d\theta_{c}$, where c=(L,R) is the index of the contact side, *θ_c_* is the electron trajectory angle from the electron reservoir into the PCP.

Equation (4) must satisfy the Zaitsev quantum boundary conditions (BCs) for normal metal and superconductive junctions [4]. These BCs were modified and applied to a ferromagnetic contact [2,3] as follows:

(5) $$\begin{array}{c} g_{a,L\alpha }\left(0,r\right)=g_{a,R\alpha }\left(0,r\right)=g_{a,\alpha }\left(0,r\right)\;\text{for}\;k_{∥,\alpha }\leq \min\left[k_{F,\alpha }^{L},k_{F,\alpha }^{R}\right],\\ g_{a,L\alpha }\left(0,r\right)=g_{a,R\alpha }\left(0,r\right)=0\;\text{for}\;k_{∥,\alpha }>\min\left[k_{F,\alpha }^{L},k_{F,\alpha }^{R}\right] \end{array}$$

(6) $$2R_{\alpha }g_{a,\alpha }\left(0,r\right)=D_{\alpha }\left\{g_{s,L\alpha }\left(0,r\right)-g_{s,R\alpha }\left(0,r\right)\right\},$$

where *k*_||, *α*_ is the projection of the Fermi vector on the PCP; the function min [*k*_1_, *k*_2_] is the minimal value of *k*_1_ and *k*_2_; the variable *D_α_* is responsible for the quantum interference in the PCP; and $R_{\alpha }=\left(1-D_{\alpha }\right)$ are the transmission and reflection coefficients, respectively. The chemical potential difference between the contact sides $\left(\mu_{R\alpha }-\mu_{L\alpha }\right)$ is proportional to the applied voltage potential *eV*, as shown in Fig. 1, see [1]. The solution to Eq. (4) has a simpler form in the mixed coordinates’ *z* and *k* representation in the PCP using the Fourier transformation $g_{s(a),c}\left(z,r\right)=\frac{1}{{\left(2\pi \right)}^{2}}\int dk^{2}g_{s(a),c}\left(z,k\right)e^{-i\;\mathrm{kr}}$, thus yielding

(7) $$\begin{array}{c} \frac{\partial^{2}g_{s}\left(z,k\right)}{\partial z^{2}}-\kappa^{2}g_{s}\left(z,k\right)+\kappa l_{z}^{-1}\langle g_{s}\left(z,k\right)\rangle =0,\\ \frac{\partial g_{s}\left(z,k\right)}{\partial z}=-\kappa g_{a}\left(z,k\right), \end{array}$$

where $\kappa =\left[\left(1-i\;kl_{∥}\right)\right]l_{z}^{-1}$. The *c* and *α* indices were omitted here for brevity. The homogeneous equation $\frac{\partial^{2}g_{s}\left(z,k\right)}{\partial z^{2}}-\kappa^{2}g_{s}\left(z,k\right)=0$ has a general solution as follows:

(8) $$g_{s}\left(z,k\right)=C_{1}e^{\kappa z}+C_{2}e^{-\kappa z}+C_{0},$$

where *C*_1, 2_ and *C*_0_ are constants.

The exact analytical view of solution Eq. (7) was found in previous studies [5], [6], [7] as follows:

(9) $$g_{s}\left(z,k\right)=g_{a}\left(z,k\right)\text{sgn}\left(z\right)+\frac{1}{l_{z}}\int_{-\infty }^{+\infty }e^{-\kappa \;\left|\;\xi -z\;\right|}\langle g_{s}\left(\xi ,k\right)\rangle d\xi +C.$$

Eq. (9) is determined for the left (*L*) and right (*R*) half spaces of the junction:

(10) $$g_{sL}\left(z,k\right)=-g_{aL}\left(z,k\right)+\frac{1}{l_{zL}}\int_{-\infty }^{z}e^{-\kappa_{L}\left(z-\xi \right)}{\langle g_{sL}\left(\xi ,k\right)\rangle }_{\theta_{L}}d\xi +C,$$

(11) $$g_{sR}\left(z,k\right)=g_{aR}\left(z,k\right)+\frac{1}{l_{zR}}\int_{z}^{\infty }e^{-\kappa_{R}\left(\xi -z\right)}{\langle g_{sR}\left(\xi ,k\right)\rangle }_{\theta_{R}}d\xi +C.$$

Expanding *g_sR_*(*ξ*, **k**), which is a part of the integrand in Eq. (11) in a series along the quasi-classical trajectory for *ξ* → *z*, obtains:

(12) $$g_{sR}\left(\xi ,k\right)\approx g_{sR}\left(z,k\right)+{\frac{\partial g_{sR}\left(\xi ,k\right)}{\partial \xi }|}_{\xi =z}\left(\xi -z\right)+O\left[{\left(\xi -z\right)}^{2}\right],$$

where the derivative $\frac{\partial \;g_{sR}\left(\xi ,k\right)}{\partial \xi }$ can be replaced by

(13) $$\frac{\partial g_{sR}\left(z,k\right)}{\partial z}\;=-\kappa_{R}g_{aR}\left(z,k\right),$$

which is nothing but the second equation in Eq. (7). Thus, with η≡(ξ−z):

(14) $$g_{sR}\left(\xi ,k\right)\approx g_{sR}\left(z,k\right)-\kappa_{R}\eta g_{aR}\left(z,k\right).$$

In the right-hand side of Eq. (14), the combination $\left(\xi -z\right)g_{aR}\left(z,k\right)\equiv \eta g_{aR}\left(z,k\right)$ can be expressed from Eq. (11) multiplying both of its sides by *κ_R_*. Furthermore, keeping only the first leading term from Eq. (12) in the integrand of the second term in Eq. (11), transforms into:

$$\kappa_{R}g_{sR}\left(z,k\right)=\kappa_{R}g_{aR}\left(z,k\right)+\frac{\kappa_{R}}{l_{zR}}\int_{z}^{\infty }e^{-\kappa_{R}\left(\xi -z\right)}{\langle g_{sR}\left(z,k\right)\rangle }_{\theta_{R}}d\xi .$$

The average GF ${\langle g_{sR}\left(z,k\right)\rangle }_{\theta_{R}}$ can be removed from the integral as independent of *ξ*,

(15) $$\kappa_{R}g_{sR}\left(z,k\right)=\kappa_{R}g_{aR}\left(z,k\right)+\frac{1}{l_{zR}}{\langle g_{sR}\left(z,k\right)\rangle }_{\theta_{R}},$$

since $\int_{z}^{\infty }e^{-\kappa_{R}\left(\xi -z\right)}d\xi =1/\kappa_{R}$. Then Eq. (15) gives

(16) $${\langle g_{sR}\left(z,k\right)\rangle }_{\theta_{R}}=\kappa_{R}l_{zR}\left[g_{sR}\left(z,k\right)-g_{aR}\left(z,k\right)\right].$$

Using it as a part of the self-consistent loop for Eq. (11), replacing the integrand ${\langle g_{sR}\left(\xi ,k\right)\rangle }_{\theta_{R}}$ with Eq. (16) obtains:

(17) $$g_{sR}\left(z,k\right)=g_{aR}\left(z,k\right)+{ℑ}_{1}-{ℑ}_{2}+C,$$

where integrals of the first and second types, ℑ_1(2)_, are determined as ${ℑ}_{1}=\int_{z}^{\infty }e^{-\kappa_{R}\left(\xi -z\right)}\kappa_{R}g_{sR}\left(\xi ,k\right)d\xi$ and ${ℑ}_{2}=\int_{z}^{\infty }e^{-\kappa_{R}\left(\xi -z\right)}\kappa_{R}g_{aR}\left(\xi ,k\right)d\xi$, respectively. Using the second equation in Eq. (7), substituting back $\frac{\partial \;g_{sR}\left(\xi ,k\right)}{\partial \xi }$ instead of *g_aR_*(*ξ*, **k**) and integrating the third term by parts, it is possible to obtain terms with ℑ_1_ and *g_sR_*(*z*, **k**) instead of ℑ_2_. To close the self-consistent loop for ℑ_1_, it is important to use the second-order term for *g_sR_*(*ξ*, **k**) in Eq. (12) (to further develop the ℑ_1_ term):

(18) $$O\left[{\left(\xi -z\right)}^{2}\right]\approx \frac{1}{2}\frac{\partial^{2}g_{sR}\left(z,k\right)}{\partial^{2}z}\eta^{2}+O\left[\eta^{3}\right]=\frac{1}{2}\left(\kappa_{R}^{2}g_{sR}\left(z,k\right)-\frac{\kappa_{R}}{l_{zR}}\langle g_{sR}\left(z,k\right)\rangle \right)\eta^{2}+O\left[\eta^{3}\right],$$

where the second derivative is replaced according to the first equation in Eq. (7). As a result, the substitution of the series expansion including the second-order term for *g_sR_*(*ξ*, **k**) in ℑ_1_ again provides the terms with *g_sR_* (*z*,**k**), ℑ_2_, and a new type of integral:

(19) $$\begin{array}{c} {ℑ}_{3}=-\frac{1}{2}\int_{z}^{\infty }e^{-\kappa_{R}\left(\xi -z\right)}\frac{\kappa_{R}^{2}}{l_{zR}}\langle g_{sR}\left(z,k\right)\rangle {\left(\xi -z\right)}^{2}d\xi =\\ \;\;\;\;\;\;\;\;\;\;\;\;\;\;\;\;\;\;\;\;\;\;\;\;\;\;\;\;\;\;=\int_{z}^{\infty }e^{-\kappa_{R}\left(\xi -z\right)}\frac{\kappa_{R}}{l_{zR}}\langle g_{sR}\left(z,k\right)\rangle \left(\xi -z\right)d\xi =\int_{0}^{\infty }e^{-\kappa_{R}\eta }\frac{\kappa_{R}}{l_{zR}}\eta \langle g_{sR}\left(z,k\right)\rangle d\eta . \end{array}$$

We consider Eqs. (10) and (11) in terms of ℑ_3_ at *z* → 0 as follows:

(20) $$g_{sR}\left(z,k\right)=g_{aR}\left(z,k\right)+\frac{{\tilde{c}}_{R}}{l_{zR}}\int_{0}^{\infty }e^{-\kappa_{R}\eta }\eta \kappa_{R}{\langle g_{sR}\left(0,k\right)\rangle }_{\theta_{R}}d\eta +C.$$

A similar procedure gives the solution to the left side:

(21) $$g_{sL}\left(z,k\right)=-g_{aL}\left(z,k\right)+\frac{{\tilde{c}}_{L}}{l_{zL}}\int_{0}^{\infty }e^{-\kappa_{L}\eta }\eta \kappa_{L}{\langle g_{sL}\left(0,k\right)\rangle }_{\theta_{L}}d\eta +C.$$

Of note, a similar view of Eqs. (20) and (21) was found in [7]; see Eqs. (19) and (20) in [7]. However, the solution in [7] was only a supposition, without mathematical proof. The proof, Eqs. (12)-(19), was found later, motivating the authors of this dataset publication. The numerical factor ${\tilde{c}}_{R(L)}$ is responsible for collecting the numerous terms from the series expansion, where the simplified approach for the best match between the Sharvin ballistic and Maxwell diffusive limits gives the condition ${\tilde{c}}_{R(L)}=1.0$. Eqs. (20) and (21) generate more accurate solutions in comparison with the previous theoretical approaches [2,5,6]. For the next step, the system of equations for ${\langle g_{s,c}\rangle }_{\theta_{c}}$ was obtained by additional averaging of both sides of the equation by a solid angle:

(22) $${\langle g_{sL}\rangle }_{\theta_{L}}=-{\langle g_{aL}\rangle }_{\theta_{L}}+{\langle g_{sL}\rangle }_{\theta_{L}}{\tilde{c}}_{L}\int_{0}^{\infty }{\langle \frac{\eta \kappa_{L}}{l_{zL}}e^{-\kappa_{L}\eta }\rangle }_{\theta_{L}}d\eta ,$$

(23) $${\langle g_{sR}\rangle }_{\theta_{R}}={\langle g_{aR}\rangle }_{\theta_{R}}+{\langle g_{sR}\rangle }_{\theta_{R}}{\tilde{c}}_{R}\int_{0}^{\infty }{\langle \frac{\eta \kappa_{R}}{l_{zR}}e^{-\kappa_{R}\eta }\rangle }_{\theta_{R}}d\eta ,$$

where $g_{sR}=g_{s,R}\left(z>0,k\right)$ and $g_{sL}=g_{s,L}\left(z<0,k\right)$. The solution to the equations is as follows:

(24) $${\langle g_{s,c}\left(z,k\right)\rangle }_{\theta_{c}}=\frac{\text{sgn}(z)}{\left(1-\lambda_{c}\right)}{\langle g_{a,c}\left(z,k,\varepsilon \right)\rangle }_{\theta_{c}},$$

where

$$\lambda_{\;c}={\tilde{c}}_{c}\int_{0}^{\infty }{\langle \frac{\eta \kappa_{c}}{l_{z,c}}e^{-\kappa_{c}\eta }\rangle }_{\theta_{c}}d\eta =\frac{{\tilde{c}}_{c}}{1+k^{2}l_{c}^{2}}.$$

The following integral without indexes *c* and *α* is:

$\lambda =\tilde{c}\int_{0}^{\infty }{\langle \frac{\eta \kappa }{lx}e^{-\kappa \eta }\rangle }_{\theta ,\varphi }d\eta$, where *x* ≡ cos (*θ*) and $l_{z}=l\cdot x$, and $\kappa =\left[1-i(kl_{∥})\right]/l_{z}={\left(lx\right)}^{-1}-ik\sqrt{\left(1-x^{2}\right)}\cos\left(\varphi \right)/x$.

After substitution, it is expressed as follows:$\lambda =\tilde{c}\int_{0}^{\infty }{\langle l_{z}^{-1}\eta \kappa e^{-\kappa \eta }\rangle }_{\theta ,\varphi }d\eta =\tilde{c}\int_{0}^{\infty }{\langle {\left(lx\right)}^{-2}\eta e^{-\kappa \eta }\rangle }_{\theta ,\varphi }d\eta -ik\tilde{c}\int_{0}^{\infty }{\langle lx^{-2}\eta \sqrt{\left(x^{2}-1\right)}\;e^{-\kappa \eta }\cos\left(\varphi \right)\rangle }_{\theta ,\varphi }d\eta$.

The second term is equal to zero with the approximation $\kappa ={\left(lx\right)}^{-1}$:

$$\int_{0}^{\infty }{\langle lx^{-2}\eta \sqrt{\left(x^{2}-1\right)}\;e^{-\kappa \eta }\cos\left(\varphi \right)\rangle }_{\theta ,\varphi }d\eta =\frac{1}{2\pi }\int_{0}^{1}dx\int_{0}^{\infty }d\eta \int_{0}^{2\pi }lx^{-2}\eta \sqrt{\left(x^{2}-1\right)}\;e^{-\kappa \eta }\cos\left(\varphi \right)\;d\varphi =0.$$

The first term can be evaluated as follows:

$$\begin{array}{c} \lambda =\tilde{c}\int_{0}^{\infty }{\langle {\left(lx\right)}^{-2}\eta \;e^{-\kappa \eta }\rangle }_{\theta ,\;\varphi }d\eta =\frac{\tilde{c}}{2\pi }\int_{0}^{1}dx\int_{0}^{\infty }d\eta \;\;{\left(lx\right)}^{-2}\eta e^{-\eta /(lx)}\int_{0}^{2\pi }d\varphi \;e^{-\left[i\cdot \kappa \;\eta \;\cos\left(\varphi \right)\;\sqrt{x^{-2}-1}\right]}\;=\\ \;\;\;\;\;\;\;\;\;\;\;\;\;\;\;\;\;\;\;=\tilde{c}\int_{0}^{1}dx\int_{0}^{\infty }d\eta {\left(lx\right)}^{-2}\eta \;e^{-\eta /(lx)}J_{0}\left(k\eta \;x^{-1}\sqrt{1-x^{2}}\right)=\tilde{c}\int_{0}^{1}{\left[1+k^{2}l^{2}\left(1-x^{2}\right)\right]}^{-3/2}dx=\frac{\tilde{c}}{1+k^{2}l^{2}} \end{array}$$

Thus, $\lambda_{\;c,\alpha }={\tilde{c}}_{c}{\left[1+k^{2}l_{c,\alpha }^{2}\right]}^{-1}$. Of note, the derived *λ_c_* as a function of *k* is crucially different from the previous approaches [2,5,6], where $\lambda_{c}={\left(kl_{c}\right)}^{-1}\arctan\left(kl_{c}\right)$ instead.

The GF *g_s_*(*z*, **k**) is accurately defined up to a constant. This constant is equal to the equilibrium GF, $C_{R}=g_{s,R}^{eq}\left(\varepsilon \right)=\text{tanh}\left(\frac{\varepsilon }{2k_{B}T}\right)$ and $C_{L}=g_{s,L}^{eq}\left(\varepsilon \right)=\text{tanh}\left(\frac{\varepsilon -eV}{2k_{B}T}\right)$, where ɛ is the electron energy, and thus the general GF *f_s_*(*z*, **k**, ɛ) has to be redefined in the energy representation:

(25) $$f_{s}\left(z,k,\varepsilon \right)\equiv g_{s}^{eq}\left(\varepsilon \right)\Gamma \left(k\right)+g_{s}\left(z,k\right),$$

where $\Gamma \left(k\right)=\int_{0}^{a}dr\int_{0}^{2\pi }re^{\left(i\cdot \mathrm{kr}\right)}\;d\phi =\frac{2\pi a}{k}\;J_{1}\left(ka\right)$ is obtained due to integration of the constant. As a result, Eq. (25) is rewritten as:

(26) $$f_{s,c}\left(z,k\right)=g_{s,c}^{eq}\left(\varepsilon \right)\Gamma \left(k\right)\pm g_{a,c}\left(z,k,\varepsilon \right)+{\langle g_{s,c}\left(z,k\right)\rangle }_{\theta_{c}}\frac{{\tilde{c}}_{c}}{\;l_{z,c}}\int_{0}^{\infty }\;\kappa_{c}\eta \;e^{-\kappa_{c}\eta }\;d\eta ,$$

where the lower sign (−) is for c=L and (+) for c=R. The next step represents the substitution of Eq. (24) into Eq. (26) and further substitution into redefined Eq. (6) in the following form:

(27) $$2\left(1-D_{\alpha }\right)g_{a,\alpha }\left(0,k\right)=D_{\alpha }\left\{f_{s,L\alpha }\left(0,k\right)-f_{s,R\alpha }\left(0,k\right)\right\}.$$

Of note, Eqs. (26) and (27) are formulated in different ways than in [7], providing a clarified mathematical description of the model.

Asymmetric GF, *g_a_*(*z* → 0, **k**, ɛ), is expressed as follows, keeping in mind the first equation in Eq. (5):

(28) $$\begin{array}{c} g_{a}\left(0,k,\varepsilon \right)=-\frac{1}{2}D\left[\tanh\left(\frac{\varepsilon }{2k_{B}T}\right)-\tanh\left(\frac{\varepsilon -eV}{2k_{B}T}\right)\right]\Gamma \left(k\right)-\\ \;\;\;\;\;\;\;\;\;\;\;\;\;\;\;\;\;\;\;\;\;\;\;\;\;\;\;\;\;\;\;\;\;-\frac{1}{2}\frac{{\langle g_{aL}\rangle }_{\theta_{L}}}{1-\lambda_{L}}\int_{0}^{\infty }D{\left(l_{L}x_{L}\right)}^{-2}\eta \;e^{-\kappa_{L}\eta }d\eta -\frac{1}{2}\frac{{\langle g_{aR}\rangle }_{\theta_{R}}}{1-\lambda_{R}}\int_{0}^{\infty }D{\left(l_{R}x_{R}\right)}^{-2}\eta \;e^{-\kappa_{R}\eta }\;d\eta , \end{array}$$

where the *α* index is omitted.

As a next step, the system of two equations was derived, averaging over the right and then left solid angles of Eq. (28), respectively. The unknown variables ${\langle g_{aL}\rangle }_{\theta_{L}}$ and ${\langle g_{aR}\rangle }_{\theta_{R}}$ are found utilizing BCs Eq. (5). The solutions for ${\langle g_{aL}\rangle }_{\theta_{L}}$ and ${\langle g_{aR}\rangle }_{\theta_{R}}$ are substituted into Eq. (28). The derived *g_a_*(*z*, **k**, ɛ) determines the requested current density in Eq. (1). The integration over ɛ leads to a spin-resolved current for the PC in Eq. (3), where an average over *θ_L_* is chosen for convenience due to the assumption of the electron flow from the left into the right side of the PC:

(29) $$I_{\alpha }^{z}=\frac{e^{2}k_{\text{min}}^{2}a^{2}V}{2\pi \hbar }\int_{0}^{\infty }\;\frac{J_{1}^{2}\left(ka\right)}{k}F_{\alpha }\left(k\right)\;dk,$$

where

$$F_{\alpha }\left(k\right)={\langle x_{L,\alpha }D_{\alpha }\rangle }_{\theta_{L}}-\left(N_{1}{\langle x_{L,\alpha }W_{L}\rangle }_{\theta_{L}}+N_{2}{\langle x_{L,\alpha }W_{R}\rangle }_{\theta_{L}}\right),$$

$$N_{1}=\left\{{\langle D_{\alpha }\rangle }_{\theta_{L,\alpha }}\left[2\left(1-\lambda_{R}\right)+\lambda_{2}\right]-{\langle D_{\alpha }\rangle }_{\theta_{R,\alpha }}\lambda_{4}\right\}\Delta^{-1},$$

$$N_{2}=\left\{{\langle D_{\alpha }\rangle }_{\theta_{R,\alpha }}\left[2\left(1-\lambda_{L}\right)+\lambda_{1}\right]-{\langle D_{\alpha }\rangle }_{\theta_{L,\alpha }}\lambda_{3}\right\}\Delta^{-1},$$

$$\Delta =4\left(1-\lambda_{L}\right)\left(1-\lambda_{R}\right)+2\left[\lambda_{1}\left(1-\lambda_{R}\right)+\lambda_{2}\left(1-\lambda_{L}\right)\right]-\lambda_{3}\lambda_{4}+\lambda_{1}\lambda_{2},$$

where

$$\lambda_{1}={\langle \frac{D_{\alpha }}{{\left[1+{\left(kl_{L,\alpha }\right)}^{2}\left(1-x_{L,\alpha }^{2}\right)\right]}^{\;3/2}}\rangle }_{\theta_{L,\alpha }},\;\lambda_{2}={\langle \frac{\delta_{\alpha }x_{L,\alpha }D_{\alpha }}{\sqrt{x_{L,\alpha }^{2}+x_{T}^{2}}\;{\left[1+{\left(kl_{R,\alpha }\delta_{\alpha }\right)}^{2}\left(1-x_{L,\alpha }^{2}\right)\right]}^{3/2}}\rangle }_{\theta_{L,\alpha }},$$

$$\lambda_{3}={\langle \frac{\delta_{\alpha }x_{L,\alpha }D_{\alpha }}{\sqrt{x_{L,\alpha }^{2}+x_{T}^{2}}{\left[1+{\left(kl_{L,\alpha }\right)}^{2}\left(1-x_{L,\alpha }^{2}\right)\right]}^{\;3/2}}\rangle }_{\theta_{L,\alpha }},\;\lambda_{4}={\langle \frac{D_{\alpha }}{{\left[1+{\left(kl_{R,\alpha }\delta_{\alpha }\right)}^{2}\left(1-x_{L,\alpha }^{2}\right)\right]}^{\;3/2}}\rangle }_{\theta_{L,\alpha }},$$

$${\langle x_{L,\alpha }W_{L}\rangle }_{\theta_{L}}=\int_{0}^{\infty }{\langle x_{L,\alpha }D_{\alpha }\frac{\eta e^{-\kappa_{L,\alpha }\;\eta }}{l_{L,\alpha }^{2}}\rangle }_{\theta_{L,\alpha }}d\eta \;\;=\int_{\tilde{x}}^{1}\frac{x_{L,\alpha }D_{\alpha }}{{\left[1+{\left(kl_{L,\alpha }\right)}^{2}\left(1-x_{L,\alpha }^{2}\right)\right]}^{\;3/2}}dx_{L,\alpha },$$

$${\langle x_{L,\alpha }W_{R}\rangle }_{\theta_{L}}=\int_{0}^{\infty }{\langle x_{L,\alpha }D_{\alpha }\frac{\eta e^{-\kappa_{R,\alpha }\eta }}{{\left(l_{R,\alpha }x_{R,\alpha }\right)}^{2}}\rangle }_{\theta_{L,\alpha }}d\eta \;\;=\int_{\tilde{x}}^{1}\frac{x_{L,\alpha }D_{\alpha }}{{\left[1+{\left(kl_{R,\alpha }\delta_{\alpha }\right)}^{2}\left(1-x_{L,\alpha }^{2}\right)\right]}^{\;3/2}}dx_{L,\alpha }.$$

The additional definitions are $k_{\text{min}}=\text{min}\left[k_{F,\alpha }^{L},k_{F,\alpha }^{R}\right]$, $\delta_{\alpha }=k_{F,\alpha }^{L}/k_{\alpha }^{R}\left(V\right)$ with $k_{\alpha }^{R}\left(V\right)=\sqrt{{\left(k_{F\alpha }^{R}\right)}^{2}+\left(2m_{R}e/\hbar^{2}\right)V}$, the threshold value $x_{T}=\sqrt{\left(1-\delta_{\alpha }^{2}\right)/\delta_{\alpha }^{2}}$, and ${\langle D_{\alpha }\rangle }_{\theta_{R,\alpha }}={\langle \frac{\delta_{\alpha }x_{L,\alpha }D_{\alpha }}{\sqrt{x_{L,\alpha }^{2}+x_{T}^{2}}}\rangle }_{\theta_{L,\alpha }}$.

The BCs in Eq. (5) satisfy the conservation of *k*_∥,*α*_, where $k_{∥,\alpha }=k_{F,\alpha }^{L}\sin\left(\theta_{L,\alpha }\right)=k_{\alpha }^{R}\left(V\right)\sin\left(\theta_{R,\alpha }\right)$; therefore, the lower integral limit $\tilde{x}$ in ${\langle ...\rangle }_{\theta_{L,\;\alpha }}\equiv \int_{\tilde{x}}^{1}\left(...\right)dx_{L,\;\alpha }$ is described as follows: when an electron moves from the state with $k_{F,\alpha }^{L}$ to $k_{\alpha }^{R}$ and $k_{F,\alpha }^{L}\leq k_{\alpha }^{R}$ then $\tilde{x}=0$ for *δ_α_* ≤ 1; otherwise, when $k_{F,\alpha }^{L}>k_{\alpha }^{R}$, the limit is $\tilde{x}=\sqrt{\left(\delta_{\alpha }^{2}-1\right)/\delta_{\alpha }^{2}}$. These conditions for $\tilde{x}$ can be combined into $\tilde{x}=\text{Re}\left[\sqrt{\left(\delta_{\alpha }^{2}-1\right)/\delta_{\alpha }^{2}}\right]$. The condition for the negative applied voltage *V* can easily be obtained using the symmetry of the system: the main parameters of the contacting PC sides must be redefined as $k_{F,\alpha }^{L}\to k_{\alpha }^{R}\left(V\right)$, $k_{\alpha }^{R}\left(V\right)\to k_{F,\alpha }^{L}$. It is assumed that the left side is grounded and its conduction band minimum does not move with *V*. Otherwise, the following conditions must be fulfilled, modifying both $k_{\alpha }^{L}$ and $k_{\alpha }^{R}$ with *V*:

$$k_{\alpha }^{L}\left(V\right)=\sqrt{{\left(k_{F\alpha }^{L}\right)}^{2}-\left(2m_{L}e/\hbar^{2}\right)V/2},$$

$$k_{\alpha }^{R}\left(V\right)=\sqrt{{\left(k_{F\alpha }^{R}\right)}^{2}+\left(2m_{R}e/\hbar^{2}\right)V/2}\;\text{and}\;\delta_{\alpha }=k_{\alpha }^{L}\left(V\right)/k_{\alpha }^{R}\left(V\right).$$

This theory has considerable generality and applicability: it works with the spin-resolved conductance model of nanoscale objects: nanocontacts, single or multi-barrier magnetic/non-magnetic tunnel junctions, ferroelectric tunnel junctions, and DW resistance in nanocontacts and nanowires.

### 2.2 Application 1. Domain wall resistance in a magnetic PC

As a first example of the model's application, we consider the resistance behavior of a magnetic point contact (MPC) on its diameter, as shown in Fig. 1. The resistance of the MPC increases due to additional electron scattering on a short magnetic DW [8] present in a contact area when the magnetizations on the left and right sides are opposite. The MPC's resistance with a single DW was simulated by $R_{\text{DW}\;}=V/\left(I_{↑}^{\text{DW}}+I_{↓}^{\text{DW}}\right)$ where $I_{↓,↑}^{\text{DW}}$ was derived using Eq. (29) with a sloped potential approach utilizing an expression for a transmission *D*_↑,↓_ according to Ref. [5]. When the magnetizations were in the parallel direction, the MPC's resistance (*R*_0_) was obtained using Eq. (29) for $D_{↑,↓}=1.0$. The raw data for Δ*R, R*_0_, and *R*_DW_ resistances *vs* the PC's diameter, a Fortran code, and a data conversion program are available in [9].

### 2.3 Application 2. Conductance of non-magnetic PCs

As a second example, a non-magnetic and symmetric PC model of the *I-V* curve is considered. The total current $I^{z}=I_{↑}^{z}+I_{↓}^{z}$, where $I_{↓,↑}^{z}$ of general expression in Eq. (29) is reduced to the form of Eq. (30) to describe symmetric non-magnetic PC using the following relations:

$$D_{↑,↓}=1.0,\;l_{L,\alpha }=l_{R,\alpha }=l,\;k_{F,\alpha }^{L}=k_{F,\alpha }^{R}=k_{F},\;\tilde{x}=0,\;\lambda_{L(R)}=\frac{{\tilde{c}}_{L(R)}}{1+{\left(kl\right)}^{2}}.$$

Thus,

$$F_{\alpha }\left(k\right)={\langle x_{L}\rangle }_{\theta_{L}}-\left(N_{1}{\langle x_{L}W_{L}\rangle }_{\theta_{L}}+N_{2}{\langle x_{L}W_{R}\rangle }_{\theta_{L}}\right)=\frac{1}{2}-{\langle x_{L}W_{L}\rangle }_{\theta_{L}}\left(N_{1}+N_{2}\right),$$

where

$${\langle x_{L}W_{L}\rangle }_{\theta_{L}}={\langle x_{L}W_{R}\rangle }_{\theta_{L}}=\int_{0}^{1}\frac{x\;dx}{{\left[1+{\left(kl\right)}^{2}\left(1-x^{2}\right)\right]}^{\;3/2}}=\frac{1}{1+k^{2}l^{2}+\sqrt{1+k^{2}l^{2}}}.$$

When *D_α_* is independent of *V*, the conductance $G=\frac{dI}{dV}$ is simplified to $G=(I_{↑}^{z}+I_{↓}^{z})/V$. The solution of the conductance in the non-magnetic limit becomes:

$$G=\frac{G_{0}k_{F}^{2}a^{2}}{2}\left[\int_{0}^{\infty }\frac{J_{1}^{2}\left(ka\right)}{k}F_{↑}\left(k\right)\;dk+\int_{0}^{\infty }\frac{J_{1}^{2}\left(ka\right)}{k}F_{↓}\left(k\right)\;dk\right],$$

where *G*_0_ is a conductance quantum. After substitutions $F_{↓}\left(k\right)=F_{↑}\left(k\right)$, ${\tilde{c}}_{L(R)}=1.0$, $N_{1}=N_{2}$, y=ka, and $\int_{0}^{\infty }\frac{J_{1}^{2}\left(y\right)}{y}dy=\frac{1}{2}$, the expression for the conductance is as follows:

(30) $$G=4G_{S}\left(\frac{1}{4}-\int_{0}^{\infty }\frac{dy}{y}\frac{J_{1}^{2}\left(y\right)}{1+{\left(yK\right)}^{2}+\sqrt{1+{\left(yK\right)}^{2}}}\right),$$

where $G_{S}=\frac{e^{2}a^{2}k_{F}^{2}}{4\pi \hbar }$ , and *K*is the Knudsen ratio: K=l/a=2l/d. The derived solution matches the Maxwell $G_{M}=\frac{8K}{3\pi }G_{S}$ and Sharvin *G_S_* limits only when ${\tilde{c}}_{L(R)}=1.0$, resulting in $(N_{1}+N_{2})=1.0$. The integral obtained in Eq. (30) has the following properties: $\lim_{K\to 0}\left(\int_{0}^{\infty }\frac{dy}{y}\frac{J_{1}^{2}\left(y\right)}{1+{\left(yK\right)}^{2}+\sqrt{1+{\left(yK\right)}^{2}}}\right)=\frac{1}{4}-\frac{2K}{3\pi }$ and $\lim_{K\to \infty }\left(\int_{0}^{\infty }\frac{dy}{y}\frac{J_{1}^{2}\left(y\right)}{1+{\left(yK\right)}^{2}+\sqrt{1+{\left(yK\right)}^{2}}}\right)=0$. The simplified non-magnetic case, Eq. (30), and the generalized analytical solution, Eq. (29), were used for an experimental data fitting in the original article [1]. As an example, Fig. 2 shows the conductance of the Sharvin limits, alternative Boltzmann-based [11], and present model solutions *vs* golden PC’ diameters. The program builder for Fig. 2 is enabled in Supplementary Materials. The present model curves were calculated by Eq. (30), where the upper integral limit was set to 10^4^ instead of ∞. Boltzmann-based solutions for the conductance of the golden nanocontacts were derived at $k_{F}=0.9\;{\text{Å}}^{-1}$ and $k_{F}=0.96\;{\text{Å}}^{-1}$ from the equation below:

(31) $$G=G_{S}\left(\frac{8K}{8K+3\pi \;\gamma_{\text{fit}}\left(K\right)}\right),$$

which was found using Eq. (3) and Eq. (4) in [11] by Nikolić and Allen, the fitting parameter according to their approach with 1% accuracy is $\gamma_{\text{fit}}\left(K\right)=\left(\frac{1+0.83K}{1+1.33K}\right)$. Relatively good fitting of the experimental data using Eq. (31) was found at $k_{F}=0.96\;{\text{Å}}^{-1}$for l=35nm, while the solution (30) gives the best fitting at $k_{F}=0.9\;{\text{Å}}^{-1}$for l=33.5nm, Fig. 2 (the magenta dash-dot curve almost coincides with the violet dash-dot-dot curve, respectively). Parameters for the related *k_F_* were extracted from Fig. 3(b) and Fig. 3(g) of the measured Au(111) Fermi surfaces in [12]. Detailed comparisons of the Boltzmann-based model and other solutions for symmetric non-magnetic PCs are shown in Fig. 2 in our related article [1].

## Declaration of Competing Interest

The authors declare that they have no known competing financial interests or personal relationships that have, or could be perceived to have, influenced the work reported in this article.

## Appendix Supplementary materials

Image, application 1

Image, application 2

## Footnotes

Supplementary material associated with this article can be found, in the online version, at doi:10.1016/j.dib.2020.106233

## References

[1] Useinov A., Lin H.H., Useinov N., Tagirov L. (2020). Spin-resolved electron transport in nanoscale heterojunctions. Theory and applications. J. Magn. Magn. Mater..

[2] Tagirov L.R., Vodopyanov B.P., Efetov K.B. (2001). Ballistic *versus* diffusive magnetoresistance of a magnetic point contact. Phys. Rev. B.

[3] Tagirov L.R., Garcia N. (2007). Quasiclassical boundary conditions for a contact of two metals. Superlattices Microstruct..

[4] Zaitsev A.V. (1984). Quasiclassical equations of the theory of superconductivity for contiguous metals and the properties of constricted microcontacts. Sov. Phys. JETP.

[5] Useinov A.N., Deminov R.G., Tagirov L.R., Pan G. (2007). Giant magnetoresistance in nanoscale ferromagnetic heterocontacts. J. Phys. Condens. Matter.

[6] Useinov N. (2015). Semiclassical Green's functions of magnetic point contacts. Theor. Math. Phys..

[7] A. Useinov, H.H. Lin, N. Useinov, L. Tagirov, Quantum point contact: a case of spin-resolved electron transport, ArXiv (2017) 1710.00496v1. https://arxiv.org/abs/1710.00496

[8] Bruno P. (1999). Geometrically constrained magnetic wall. Phys. Rev. Lett..

[9] https://data.mendeley.com/datasets/t868bx922b/4

[10] Jensen B.D., Huang K., Chow L.L.W., Kurabayashi K. (2005). Low-force contact heating and softening using micromechanical switches in diffusive-ballistic electron-transport transition. Appl. Phys. Lett..

[11] Nikolić B., Allen B.P. (1999). Electron transport through a circular constriction. Phys. Rev. B.

[12] Matsui F., Makita S., Matsuda H., Ueba T. (2020). Bulk and surface band dispersion mapping of the Au(111) surface by acceptance-cone tunable PES system. E-J. Surf. Sci. Nanotechnol..

