# Supplementary material for: Mathematical description data: Spin-resolved electron transport in nanoscale heterojunctions: Theory and applications
Source: Data Brief. 2020 Sep 1;32:106233. doi: 10.1016/j.dib.2020.106233 (PMC7494667; doi:10.1016/j.dib.2020.106233)
Supplement: Supplementary file 1 [file mmc1.pdf]

```
(* PROGRAM DATA BUILDER FOR FIG 2 in [A. Useinov et al, Data in Brief vol. 32,
  Avl. online: 1 Sept.(2020) 106233,https://doi.org/10.1016/j.jmmm.2020.166729]*)

(*Erts data: D.Erts,et al.,Phys.Rev.B 61(2000) 12725-
12727,  data is {a^2 vs Conductance }*)

In[ ]:= Exp1 = {{0.265878877`, 0.000658598368`}, {0.576070901`, 0.00106388967`},
    {0.753323486`, 0.00146918097`}, {1.01920236`, 0.00192513369`},
    {1.28508124`, 0.00265128061`}, {3.19054653`, 0.00808893892`},
    {4.16543575`, 0.00788629327`}, {4.47562777`, 0.00776808331`},
    {4.80797637`, 0.00846045595`}, {5.53914328`, 0.00817337461`},
    {5.2732644`, 0.00923726428`}, {5.07385524`, 0.00981142696`},
    {5.71639586`, 0.00952434562`}, {5.71639586`, 0.0102167183`},
    {5.38404727`, 0.0109766395`}, {7.0901034`, 0.0109090909`}, {5.98227474`, 0.0121587391`},
    {6.02658789`, 0.0121249648`}, {6.97932053`, 0.0122094005`},
    {7.73264402`, 0.0121249648`}, {9.54948301`, 0.0120743034`},
    {10.4579025`, 0.0122094005`}, {6.93500739`, 0.0131044188`},
    {7.95420975`, 0.0129355474`}, {8.53028065`, 0.0129355474`},
    {9.394387`, 0.0131381931`}, {10.0812408`, 0.0132226288`}, {10.7680945`, 0.0131044188`},
    {11.4771049`, 0.0131381931`}, {11.8537666`, 0.0129017731`},
    {6.55834564`, 0.0146918097`}, {7.51107829`, 0.0146411483`},
    {8.06499261`, 0.0142020827`}, {9.0620384`, 0.0142696313`}, {10.3028065`, 0.0139994371`},
    {10.2363368`, 0.0146411483`}, {10.6794682`, 0.0221559246`},
    {15.5760709`, 0.0224092316`}, {16.4401773`, 0.0223247959`},
    {17.0827179`, 0.0223247959`}, {17.6587888`, 0.0221559246`},
    {10.7237814`, 0.0227469744`}, {10.5243722`, 0.0234224599`}, {12.9172821`, 0.02318604`},
    {13.5155096`, 0.0231016043`}, {15, 0.02318604`}, {11.4327917`, 0.0241486068`},
    {16.3958641`, 0.0240810583`}, {17.7252585`, 0.0241148325`},
    {18.6779911`, 0.0241486068`}, {16.7060561`, 0.0251280608`},
    {19.0546529`, 0.0251787222`}, {20.7163959`, 0.0251280608`},
    {17.0827179`, 0.0266816775`}, {17.8360414`, 0.0262763862`},
    {18.1905465`, 0.0264283704`}, {18.633678`, 0.0261919505`}, {19.4756278`, 0.0261412891`},
    {20.3840473`, 0.0261919505`}, {18.3677991`, 0.0271545173`},
    {21.070901`, 0.0272051787`}, {19.5420975`, 0.02772868`}, {15.9084195`, 0.0285054883`},
    {17.2378139`, 0.0285054883`}, {17.6587888`, 0.028100197`}, {18.4121123`, 0.028218407`},
    {19.4313146`, 0.0276442443`}, {21.1373708`, 0.0271545173`},
    {19.7415066`, 0.0283872784`}, {22.5332349`, 0.0281339713`},
    {20.450517`, 0.0290289896`}, {22.88774`, 0.0291471995`}, {17.4593796`, 0.0310723332`},
    {19.5864106`, 0.0301266535`}, {21.4032496`, 0.0302110892`},
    {23.5967504`, 0.0302110892`}, {18.0354505`, 0.0327779341`}, {18.94387`, 0.0326597242`},
    {19.8079764`, 0.0318829158`}, {20.915805`, 0.0320855615`}, {21.9350074`, 0.0313425274`},
    {22.88774`, 0.0314776245`}, {26.6322009`, 0.0301266535`}, {27.6514032`, 0.0310216718`},
    {28.8257016`, 0.0310216718`}, {24.1728213`, 0.0334703068`},
    {27.5849335`, 0.0334703068`}, {27.0974889`, 0.0341626794`}, {27.6292467`, 0.034112018`},
    {28.5376662`, 0.034112018`}, {29.7784343`, 0.034112018`}, {32.0162482`, 0.0350576977`},
    {22.0901034`, 0.036070926`}, {23.1979321`, 0.0361047003`}, {28.5376662`, 0.035699409`},
    {28.1610044`, 0.0361553617`}, {29.8227474`, 0.0361047003`},
    {22.2895126`, 0.0367632986`}, {22.3559823`, 0.0377765269`},
    {25.8124077`, 0.0371685899`}, {26.8759232`, 0.0372023642`},
    {28.3161004`, 0.0377765269`}, {27.3190547`, 0.0380973825`},
    {27.7400295`, 0.0387053194`}, {37.1787297`, 0.0381480439`},
```

```
   {28.3825702`, 0.0395665635`}, {27.2082718`, 0.0400562905`},
   {28.5376662`, 0.0403771461`}, {38.042836`, 0.0400225162`}, {41.1890694`, 0.0400562905`},
   {27.4298375`, 0.0411539544`}, {28.4268833`, 0.0411201801`},
   {26.7208272`, 0.0421334084`}, {28.493353`, 0.0421671827`}, {44.3353028`, 0.0421671827`},
   {28.4268833`, 0.0430284267`}, {29.7784343`, 0.043062201`}, {29.8227474`, 0.0447340276`},
   {31.4180207`, 0.0449366732`}, {32.0605613`, 0.0454264002`},
   {32.5923191`, 0.0451393189`}, {34.9409158`, 0.0453419645`},
   {32.1713442`, 0.0462369828`}, {32.6587888`, 0.0464058542`},
   {33.3456425`, 0.0461525471`}, {34.8966027`, 0.0453419645`},
   {44.3353028`, 0.0461863214`}, {41.0339734`, 0.0481452294`},
   {46.5731167`, 0.0481790037`}, {52.0236337`, 0.0481452294`},
   {52.7104874`, 0.0508302843`}, {6.93500739`, 0.0136279201`},
   {7.75480059`, 0.013374613`}, {4.98522895`, 0.00790318041`},
   {0.64254062`, 0.00233042499`}, {29.4239291`, 0.0427075711`}};

In[]:= DataErtsVSd = Table[{2 * Sqrt[Exp1[[i, 1]]], Exp1[[i, 2]]}, {i, 1, Length[Exp1]}];
   (* {d vs Conductance }*)

In[]:= (*Jensen data from Fig. 4: B.D.Jensen,
   et al.Appl.Phys.Lett.86 (2) (2005) 023507. https://doi.org/10.1063/1.1850191 *)

In[]:= (*  data: a^2 , Conductance *)
   ExpJ = {{1.7766538447051772`, 0.0029424879634366683`},
     {2.5291034347170585`, 0.004205763567457859`},
     {4.423770221979418`, 0.007077691973423805`}, {12.490766847287857`,
      0.01968923104458315`}, {19.163930301962605`, 0.0296155884184735`},
     {25.311427831977596`, 0.03861534441799924`}, {29.167185390910163`,
      0.04454633478102982`}, {32.46148567471116`, 0.048831783807238884`},
     {38.938029674643744`, 0.0580833321682785`}, {41.52009074943175`, 0.06112416981458179`},
     {75.59828886948894`, 0.11189899128710253`}, {108.77350561679081`, 0.1519829534338806`},
     {420.00623303381576`, 0.4827827574968547`}, {424.5248515707131`, 0.4827827574968547`},
     {514.7022757513367`, 0.5684187486808067`}, {604.3198947970345`, 0.6424744745051898`},
     {724.8906012173986`, 0.6490649680411444`}, {604.3198947970345`, 0.7487558560382689`},
     {888.3269282121154`, 0.8997491733239559`}, {1112.1617386427247`, 1.0784365762169579`},
     {2653.1887410831027`, 1.9592217306717763`}};

   DataJenVSd = Table[{2 * Sqrt[ExpJ[[i, 1]]], ExpJ[[i, 2]]}, {i, 1, Length[ExpJ]}];
   (* {d vs Conductance }*)

   (*Useinov et al. JMMM 2020, Data in Brief 2020: working Eq.(30) for non-mag PCs: *)
   (* G_0 = 2e^2/h = 7.7481*10^(-5)  is a Conductance quantum *)

   Eq30[a_, l_, kF_] := 4.0 * 7.7481 * 10^(-5) * (kF * a / 2)^2 * (1 / 4 - NIntegrate[
        (BesselJ[1, y]^2) / (y * (1 + (y * l / a)^2 + Sqrt[1 + (y * l / a)^2])), {y, 0, 10000}]);
   kF =
     9.0
    (*nm^
     -1*);

In[]:= Data38 = Table[{a^2, Eq30[a, 38.0, kF]}, {a, 0.5, 80.1, (80.1 - 0.5) / 140}];
```

In[ ]:=
```
ListLogLogPlot[{Data38, Exp1, ExpJ}, PlotRange → All,
 Joined → {True, False, False}, PlotStyle → {Green, Black, Red}, AspectRatio → 1.2]
```

Out[ ]=

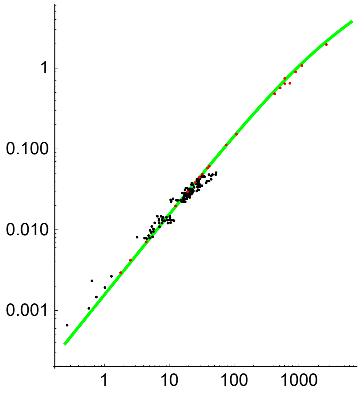

In[ ]:=

In[ ]:=
```
Export["Data38_09_vs_radius2.txt", Data38, "Table"]
```

Out[ ]= Data38_09_vs_radius2.txt

```
(* Sharvin - Ballistic Conuctance calculations *)
```

```
kF = 9.0;  (* Units: [1/nm] *)
SRSP[a_, kF_] := 7.7481 * 10^(-5) * (kF * a / 2)^2;
DataSRSPdiameter = Table[{a * 2, SRSP[a, kF]}, {a, 0.5, 180.1, (180.1 - 0.5) / 140}];
Data38d = Table[{a * 2, Eq30[a, 38.0, kF]}, {a, 0.5, 180.1, (180.1 - 0.5) / 140}];
ListLogLogPlot[{DataJenVSd, DataSRSPdiameter, DataErtsVSd, Data38d},  PlotRange → All,
 Joined → {False, True, False, True}, PlotStyle → {Green, Black, Red}, AspectRatio → 1.2]
```

Out[∘]=

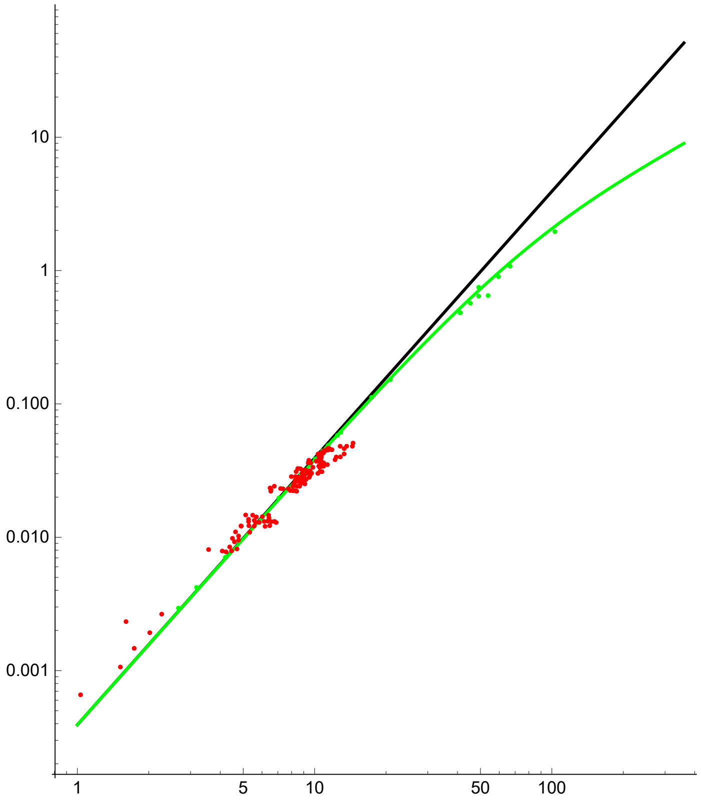

In[∘]:=

```
Export["DataSarvin_vs_diameter.txt", DataSRSPdiameter, "Table"]
```

Out[∘]= DataSarvin_vs_diameter.txt

```
(* Nikolic - Botzmann-based solution: Phys.Rev.B 60 (1999) 3963-3969. https://
 doi.org/10.1103/PhysRevB.60.3963*)

KK[a_, l_] := l / (a);(*  d, a and l in units: [nm] *)
Gma[a_, l_] := (1 + 0.83 * KK[a, l]) / (1 + 1.33 * KK[a, l]);
Botzm[a_, l_, k_] := SRSP[a, k] * (8.0 * KK[a, l] / (8.0 * KK[a, l] + 3.0 * Pi * Gma[a, l]));

SetT3BotzmannDiam = Table[{a * 2, Botzm[a, 38, 9.0]}, {a, 0.5, 180.1, (180.1 - 0.5) / 140}];
Export["Botzmann_vs_diameter.txt", SetT3BotzmannDiam, "Table"]
ListLogLogPlot[{Data38d, SetT3BotzmannDiam, DataErtsVSd, DataJenVSd},
 PlotRange → All, Joined → {True, True, False, False},
 PlotStyle → {Blue, Green, Black, Red}, AspectRatio → 1.2]
```

Out[ ]= Botzmann_vs_diameter.txt

Out[ ]=

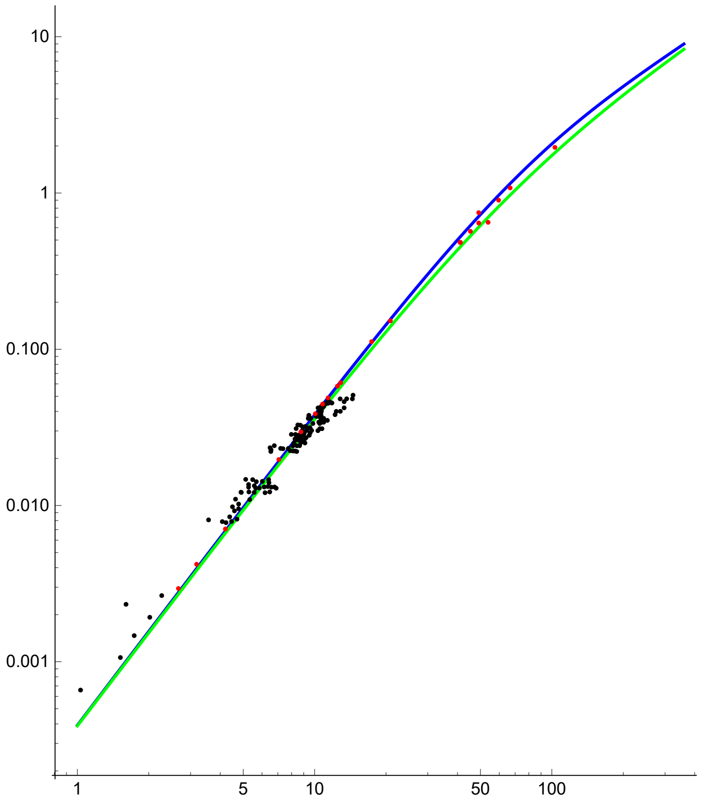

In[ ]:=

In[ ]:=

In[ ]:=
```
DataSarvinDiam1d = Table[{a * 2, SRSP[a, 9.6]}, {a, 0.5, 80.1, (80.1 - 0.5) / 140}];
DataSarvinDiam2d = Table[{a * 2, SRSP[a, 9.0]}, {a, 0.5, 80.1, (80.1 - 0.5) / 140}];
SetT3Botzmann35a096d = Table[{a * 2, Botzm[a, 35, 9.6]}, {a, 0.5, 80.1, (80.1 - 0.5) / 140}];
Data33p5a09d = Table[{a * 2, Eq30[a, 33.5, 9.0]}, {a, 0.5, 80.1, (80.1 - 0.5) / 140}];
SetT3Botzmann44a0p9d = Table[{a * 2, Botzm[a, 44, 9.0]}, {a, 0.5, 80.1, (80.1 - 0.5) / 140}];
Data27p5a0p9d = Table[{a * 2, Eq30[a, 27.5, 9.6]}, {a, 0.5, 80.1, (80.1 - 0.5) / 140}];
```

In[∘]:=

In[∘]:=
```
ListLogLogPlot[{DataErtsVSd, DataJenVSd, DataSarvinDiam1d, DataSarvinDiam2d,
  Data33p5a09d, Data27p5a0p9d, SetT3Botzmann35a096d, SetT3Botzmann44a0p9d},
 PlotRange → All, Joined → {False, False, True, True, True, True, True, True},
 PlotStyle → { Black, Red, Green, Orange, Yellow, Dashed, DotDashed}, AspectRatio → 1.0]
```

Out[∘]=

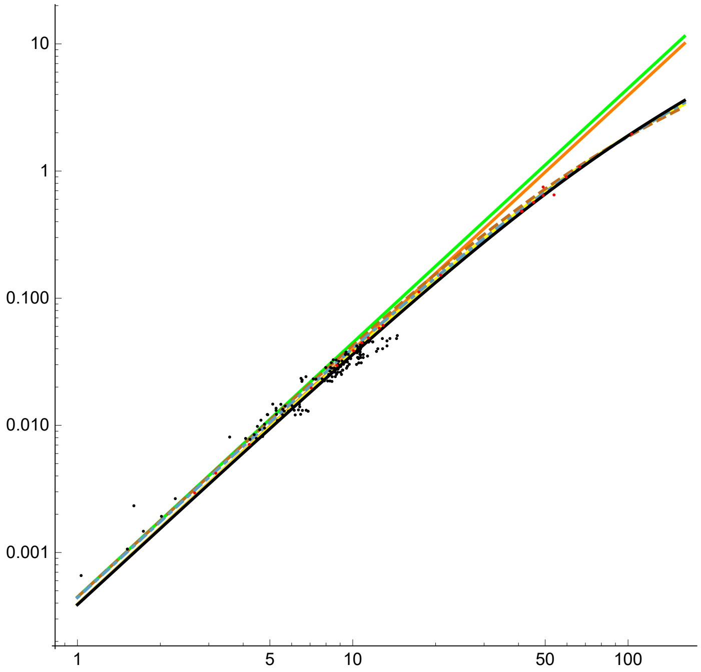

In[ ]:=
```
ListPlot[{DataErtsVSd, DataJenVSd, DataSarvinDiam1d, DataSarvinDiam2d,
  Data33p5a09d, Data27p5a0p9d, SetT3Botzmann35a096d, SetT3Botzmann44a0p9d},
 PlotRange → All, Joined → {False, False, True, True, True, True, True, True},
 PlotStyle → { Black, Red, Green, Orange, Yellow, Dashed, DotDashed}, AspectRatio → 1.0]
```

Out[ ]=

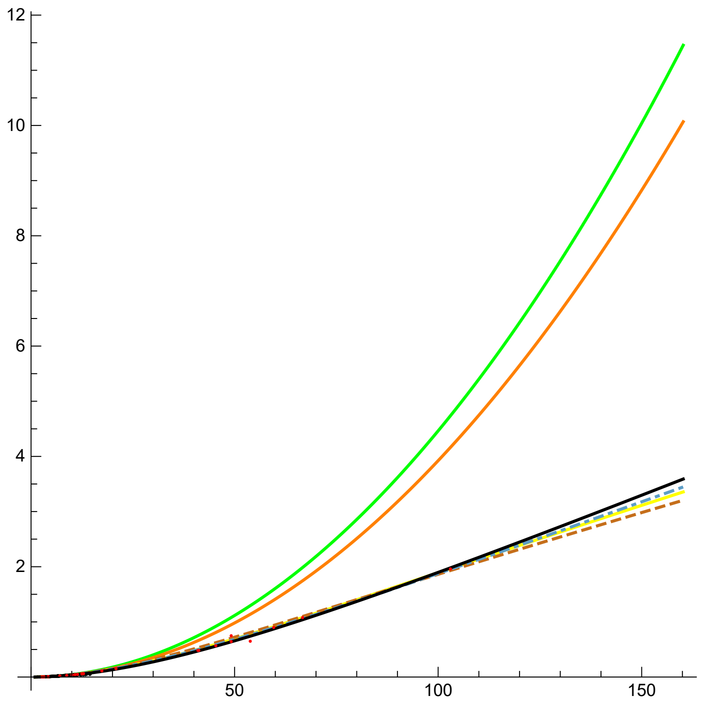

In[ ]:=
```
Export["1DataSarvinDiam1d0p96.txt", DataSarvinDiam1d, "Table"];
Export["2DataSarvinDiam2d0p9.txt", DataSarvinDiam2d, "Table"];
Export["3SetT3Botzmann35a096d.txt", SetT3Botzmann35a096d, "Table"];
Export["4Data33p5a09d.txt", Data33p5a09d, "Table"];
Export["5SetT3Botzmann44a0p9d.txt", SetT3Botzmann44a0p9d, "Table"];
Export["6Data27p5a0p9d.txt", Data27p5a0p9d, "Table"];

(*see files in folder: Documents  *)
```
